# Genetic diversity of NS5A protein from hepatitis C virus genotype 3a and its relationship to therapy response

**DOI:** 10.1186/1471-2334-10-36

**Published:** 2010-02-23

**Authors:** Cíntia Bittar, Ana Carolina G Jardim, Lilian HT Yamasaki, Artur TL de Queiróz, Claudia MA Carareto, João Renato R Pinho, Isabel Maria VG de Carvalho-Mello, Paula Rahal

**Affiliations:** 1 UNESP, São Paulo State University, IBILCE, Institute of Bioscience, Language & Literature and Exact Science, Department of Biology, Rua Cristóvão Colombo, 2265 , Bairro Jardim Nazareth, CEP 15054-010, São José do Rio Preto, São Paulo, Brazil; 2 USP, São Paulo University, Rua do Matão, trav 14, n° 321, Cidade Universitária, CEP 05508-900, São Paulo, São Paulo, Brazil; 3 USP, São Paulo University, Faculty of Medicine, Department of Gastroenterology, Laboratory of Hepatology and Gastroenterology from Institute of Tropical Medicine, Av Dr Arnaldo, 455, Cerqueira César, CEP: 01246903, São Paulo, SP, Brazil; 4 Albert Einstein Israeli Hospital, Department of Clinical Pathology, Av Albert Einstein, 627/701, CEP 05652-000São Paulo, SP, Brazil; 5 Butantan Institute, Viral Immunology Laboratory, Av Vital Brasil n° 1500, CEP 05503-900, Butantã, São Paulo, SP, Brazil

## Abstract

**Background:**

The quasispecies nature of HCV may have important implications for viral persistence, pathogenicity and resistance to antiviral agents. The variability of one of the viral proteins, NS5A, is believed to be related to the response to IFN therapy, the standard treatment for infection. In this study we analyzed the quasispecies composition of NS5A protein in patients infected with HCV genotype 3a, before IFN therapy.

**Methods:**

Viral RNA was isolated from samples of 12 patients: four sustained virological responders (SVR), four non-responders (NR), and four end-of-treatment responders (ETR). cDNA was synthesized, the NS5A region was amplified and the fragments obtained were cloned. Fifteen clones from each patient were sequenced with eight primers, generating 179 contigs.

**Results:**

Higher values for substitution (either synonymous or non-synonymous) and for distance were found in the SVR group. However, the NR group showed relatively more non-synonymous mutations than the other groups, owing to the higher values of dN/dS in complete NS5A and most specific regions. Overall, NS5A protein is undergoing purifying selection, since all dN/dS ratios values are below 0.5.

**Conclusions:**

Our study provides an overview of the genetic variability of complete NS5A protein in HCV genotype 3a.

## Background

The hepatitis C virus (HCV) is among the most successful of all persistent human viruses [1]. It is estimated that the global prevalence of HCV infection is 2.2%, corresponding to about 130 million HCV-positive persons worldwide [2]. The HCV genome consists of a single-strand positive-sense RNA of approximately 9.6 Kb that contains an open reading frame coding for a polyprotein precursor of approximately 3000 residues. This precursor is cleaved by viral and host proteinases into the viral proteins: the structural protein core, E1, E2 and p7, and the nonstructural proteins NS2, NS3, NS4A, NS4B, NS5A and NS5B [3].

To date, six main HCV genotypes have been identified, which differ by about 30% in their nucleotide and amino acid sequences [4]. Genotypes 1, 2 and 3 and their subtypes have a global distribution; genotype 4 is found in Africa, genotype 5 in South Africa, and genotype 6 mainly in Asia [5]. In Brazil, Campiotto *et al*. (2005) reported the presence of genotypes 1, 2, 3, 4 and 5 [6].

The RNA-dependent RNA polymerase encoded by the NS5B gene is error-prone and lacks proofreading. As a result, base changes are introduced randomly into the viral genome [7]. Therefore, HCV replication is associated with a high mutation rate, giving rise to a mixed and changing population of mutants known as quasispecies [4,8]. The quasispecies nature of HCV may have important implications for viral persistence, pathogenicity and resistance to antiviral agents [8-11]. This is most problematic for the infected patient, because quasispecies variation confers remarkable adaptive potential on HCV and has been implicated in the evasion and control of the host response to infection and in differential sensitivity to IFN therapy. The hostile antiviral host environment may drive the proliferation of HCV "evasion variants" from a pre-existing quasispecies pool or through viral genetic adaptation [11]. However, intra-genotype analysis of this diversity in the viral genome shows different degrees of variation; regions such as the 5'UTR and the core are highly conserved, the non-structural regions 2, 3, 5b and the 3'UTR are relatively variable, while the envelope regions E1 and E2 and the NS4 and the NS5A genes exhibit the highest sequence diversity [12]. Sequence analysis of the HCV NS5A coding region has similarly identified specific domains that exhibit sequence variation associated with the outcome of IFN therapy [13].

NS5A is the nonstructural HCV protein most frequently reported to be implicated in interferon resistance. It is a pleiotropic protein, involved both in viral replication and in many interactions with cellular signaling pathways, including the interferon anti-viral pathway [12]. This study analyzed the NS5A quasispecies pattern in patients infected with HCV genotype 3 before IFN plus ribavirin therapy, with the aim of elucidating its molecular constitution and relationship to treatment response.

## Results

### Viral load

The viral load data show that all patients presented high viral loads, as expected for pre-treatment samples, ranging from log 5.76 to log 7.03. There was no correlation between viral load and treatment response (Table 1).

**Table 1:** Viral load.

Patient	Therapy response	Viral load (UI/ml)	Log	Mean	SEM*
RF 015	Sustained virological responder	693.411	5,84	5.578.634	+/- 2.062.880
RF 018	Sustained virological responder	10.717.120	7,03		
RF 059	Sustained virological responder	6.070.135	6,78		
RF 080	Sustained virological responder	4.833.870	6,68		

RF 007	Non responder	569.141	5,76	2.348.652	+/- 703.684
RF 060	Non responder	3.168.112	6,50		
RF 075	Non responder	3.735.050	6,57		
RF 145	Non responder	1.922.304	6,28		

RF 020	End of treatment responder	4.205.798	6,62	2.422.973	+/- 688.016
RF 031	End of treatment responder	919.193	5,96		
RF 109	End of treatment responder	2.600.972	6,42		
RF 119	End of treatment responder	1.965.928	6,29		

*SEM: standard error of the mean

### Sequencing

This study generated 15 contig sequences of the full NS5A region from 11 patients; 14 sequences from patient RF80 could be sequenced, totaling 179 contigs.

### Quasispecies analysis

**Figure 1:**
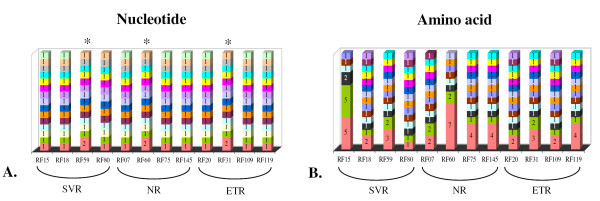
**Different nucleotide and amino acid sequences**. Graphic representation of the different nucleotide (A) and amino acid (B) sequences found in the samples from each patient. The response groups are denoted: SVR -- sustained virological responder, NR -- non-responder, ETR -- end-of-treatment responder.

Quasispecies analysis revealed that the HCV NS5A region was highly variable, as only three patients showed two identical nucleotide sequences (Figure 1A). The amino acid data showed more identical sequences owing to synonymous substitution, as can be seen when the nucleotide and amino acid patterns of patient RF15 are compared. All nucleotide sequences from this patient were different; however, the amino acid sequences showed one of the highest degrees of conservation (Figure 1B).

### Substitutions

Nucleotide substitution analysis showed that the sustained virological response (SVR) group had the highest means of nucleotide and amino acid substitutions, except for the amino acid sequences of regions NLS and V3, where the end-of-treatment response (ETR) group showed the highest values (Table 2). However, none of the differences between groups were statistically significant.

**Table 2:** Number of mutations, genetic distance, dN, dS and dN/dS.

Regions and types of treatment response	nt mutations	aa mutations	Genetic distance *ws*	Genetic distance *bs*	dS *ws*	dS *bs*	dN *ws*	dN *bs*	dN/dS *ws*	dN/dS *bs*
**NS5A**										
SVR	29.85	7.03	0.022	0.064	0.0739	0.2519*	0.0075	0.0182*	0.0876	0.0672*
NR	13.41	4.65	0.01	0.053	0.0272	0.2075*	0.0054	0.0155*	0.1365	0.0761*
ETR	26.21	5.77	0.0193	0.056	0.0506	0.2128*	0.0061	0.0164*	0.0759	0.0734*

**CRS**										
SVR	1.65	0.44	0.0203	0.055	0.1451	0.3114* ^b^	0.0192	0.0412*	0.1382* ^a^	0.1181*
NR	0.75	0.20	0.0093	0.044	0.0769	0.2303* ^c^	0.0239	0.0203*	0.2917* ^a^	0.0796*
ETR	1.25	0.26	0.0155	0.037	0.119	0.1797* ^b, ^* ^c^	0.0233	0.0223*	0.1596	0.1010*

**PKR-bd**										
SVR	5.21	1.23	0.0263	0.062	0.1308	0.2955*	0.0156	0.0171*	0.1028	0.0376* ^b^
NR	2.08	0.85	0.0105	0.055	0.042	0.2857*	0.0156	0.0147*	0.4569	0.0390* ^c^
ETR	3.55	0.73	0.0178	0.048	0.092	0.2482*	0.0089	0.0098*	0.1343	0.0336* ^b, ^* ^c^

**ISDR**										
SVR	3.04	0.60	0.0253	0.061	0.1696	0.3096*	0.0269	0.0219*	0.151	0.0472*
NR	1.17	0.40	0.0098	0.051	0.0537	0.2766*	0.022	0.0174*	0.4457	0.0521*
ETR	2.01	0.13	0.0168	0.051	0.1335	0.2814*	0.0109	0.0111*	0.1485	0.0390*

**NLS**										
SVR	0.58	0.10	0.0213	0.099	0.2658	0.4935*	0.0617	0.0598* ^a^	0.4523	0.1143*
NR	0.20	0.03	0.0075	0.068	0.1399	0.2529*	0.0541	0.0555* ^a^	0.3864	0.2166*
ETR	0.47	0.16	0.0173	0.063	0.2367	0.2102*	0.0557	0.0607	0.2663	0.2705*

**V3**										
SVR	1.69	0.76	0.0258	0.081	0.1088	0.2344*	0.0406	0.0622* ^a^	0.3415	0.1940* ^a^
NR	0.91	0.55	0.0138	0.063	0.0594	0.1246*	0.0313	0.0664* ^a, ^* ^c^	0.4752	0.4130* ^a, ^* ^c^
ETR	1.37	0.83	0.0208	0.066	0.0868	0.1817*	0.0342	0.0488* ^c^	0.4022	0.1996* ^c^

Values for number of mutations and for genetic distance, rates of synonymous substitution per synonymous site (dS), non-synonymous substitution per non-synonymous site (dN) and dN/dS ratio obtained by *ws *and *bs *analysis for each response group regarding the complete NS5A and the regions studied.

* Significant difference among all groups; p < 0.05

* ^a^Significant difference between SVR and NR; p < 0.05

* ^b ^Significant difference between SVR and ETR; p < 0.05

* ^c ^Significant difference between NR and ETR; p < 0.05

**Figure 2:**
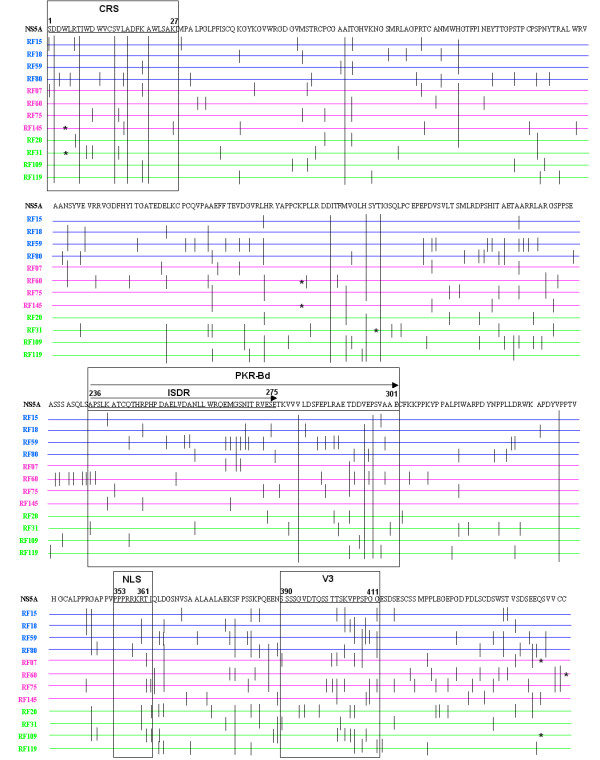
**Mutation sites**. Graphic representation of the mutation sites found in each patient, according to the reference sequence for genotype 3, NZL1 (GeneBank D17763). SVR patients are represented in blue, NR patients in pink, and ETR patients in green.

To identify the mutation sites in the sequences used in this study, they were represented graphically according to the reference sequence NZL1 (*GeneBank *D17763) (Figure 2). This representation indicates that no specific mutation could be associated with any kind of treatment response. Figure 2 also shows that some of the nucleotide mutations resulted in stop codons. Of the nine stop codons found in the 179 sequences generated in this study, the site was the same in two or more clones in seven cases. The same stop codon sites were found in NS5A amino acids 4 (RF31 and RF145), 166 (RF60 - 2 clones and RF145) and 447 (RF07 and RF109). Also, the sites where the translation stop codon was observed showed no other mutation, except for one clone from patient RF145, which showed a mutation in aa 447.

### Substitution rates

For the within-sample (*ws*) data, the mean rate of synonymous substitution per synonymous site (dS) was higher in the SVR group (Table 2). The rate of non-synonymous substitution per non-synonymous site (dN) was also higher in the SVR group, except for regions CRS and PKR-Bd. These data showed no statistically significant differences.

Between-sample (*bs*) analysis also showed a higher dS mean in the SVR group. SVR had the highest mean dN values, except for regions NLS and V3. All the *bs *data showed statistical significance, except for the dS means in CRS between SVR and ETR and between NR and ETR.

In the *ws *analysis, the dN/dS ratio was higher in the NR group, except for the NLS region, where SVR had the highest ratio. In this analysis, only the difference between SVR and NR in the CRS region was statistically significant.

The *bs *data showed higher dN/dS ratios in NR for most of NS5A; regions CRS and NLS showed higher values in SVR and ETR, respectively. Statistical significance was observed in all regions, except for PKR-Bd between SVR and NR and for region V3 between SVR and ETR.

### Genetic distances

The mean genetic distances for the complete NS5A region and the other regions studied were higher in the SVR group in either *ws *or *bs *analysis (Table 2). No statistically significant difference was found for genetic distance.

### Phylogenetic analysis

**Figure 3:**
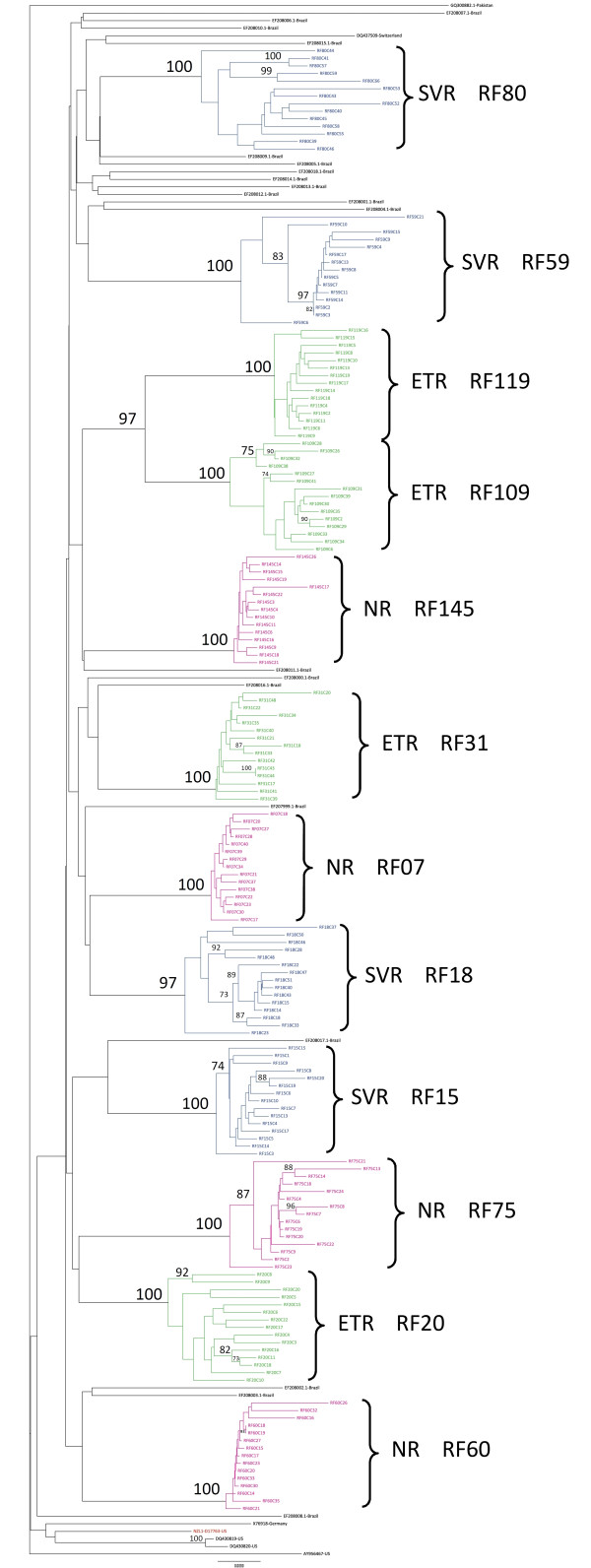
**Phylogenetic tree**. Unrooted phylogenetic tree constructed with the 179 sequences of full NS5A generated in this study, plus the reference sequence for genotype 3, NZL1 (GeneBank D17763) - Red, the six full-length NS5A sequences from genotype 3a, with country information, available in GenBank (Accession numbers: AY956467; DQ430819; DQ430820; DQ437509; X76918; GQ300882.1) and 20 Brazilian NS5A sequences with 1308 bp (Acession numbers: EF207999.1 to EF208018.1), by the distance method with the Tamura-Nei+I+G model using the neighbor-joining algorithm. Bootstrap was calculated with 1000 replications. SVR -- sustained virological responder - Blue, NR- non-responder - Pink, ETR -- end-of-treatment responder - Green.

Phylogenetic analysis was carried out using the 179 1356-bp sequences generated in this study, the reference sequence for genotype 3, NZL1 (GenBank accession number D17763), the six full-length NS5A sequences from genotype 3a, with country information, available in GenBank (Accession numbers: AY956467; DQ430819; DQ430820; DQ437509; X76918; GQ300882.1), and 20 Brazilian NS5A sequences of 1308 bp (Accession numbers: EF207999.1 to EF208018.1). The resulting phylogenetic tree is presented in Figure 3. All sequences from clones from the same patient are grouped in a monophyletic branch, with high bootstrap values (95-100%). There was no clustering in a monophyletic branch of the sequences from patients in the same treatment response group.

## Discussion

RNA viruses have high mutation rates owing to lack of proofreading of the RNA polymerase. On the one hand, these high mutation rates can be deleterious for the virus by creating non-viable strains; on the other, when the viral population is considered, they can also create a pool of potentially good mutations. The capacity of HCV to circulate as a pool of different but closely related strains, commonly called quasispecies, allows it to evolve and adapt to new environments and to overcome the challenges of infection [4,8,14-16]. The phylogenetic tree constructed in this work illustrates this characteristic and shows, for each patient, very similar but not identical strains with a monophyletic origin.

Our investigation of the genetic variability of the NS5A region from HCV 3a demonstrated the high variability of strains found in the same patient. The highest values of substitutions, either synonymous or non-synonymous, and the highest virus diversity, evaluated by genetic distance, were found in patients with sustained virological responses (SVR). Studies on genotype 1b have reported that the number of mutations in a specific region of NS5A, ISDR, could predict an SVR [17-21]. Since other studies refute these results, a meta-analysis was performed with American, European and Japanese sequences [22-30] that allowed a positive correlation to be established between mutations in the ISDR region and SVR. The same study also revealed a geographic correlation; mutations in the Japanese sequences had a higher probability of leading to SVR than mutations in European sequences. The authors suggested that geographic variation may be related to host factors. This meta-analysis included sequences of genotype 1, but no South American sequences were considered. Since the European and Japanese strains yielded different results, other populations may also present a different pattern.

Although the SVR group showed higher mutation values in the region NS5A by either *bs *or *ws *analysis in our study, the dN/dS ratio was higher in the group of non-responders (NR). Therefore, though SVR showed more mutations, NR strains had relatively more non-synonymous substitutions. Amino acid mutations are most commonly deleterious because of the changes they cause in the protein phenotype. However, some of these substitutions are neutral when the mutation does not affect protein function, and are maintained by genetic drift. The higher rates of non-synonymous mutations detected in NR patients may indicate an advantage in evading both the immune system and the treatment, since they may be able to modify epitopes so that they are no longer recognized by the immune system.

For any given genome, the mutation rate determines the ability of a virus to maintain essential information while coping with environmental changes [16,31-39]. All values obtained for comparisons between dN and dS using the dN/dS ratio were below 0.5. These findings indicate that the NS5A protein is undergoing purifying selection, which maintains protein function and therefore virus activity.

The occurrence of stop codons in the same site has been described previously in the literature. In a similar study, performed on patients infected with HCV genotype 1, two stop codons were found in the same site of the CRS region in two different patients [40]. Also, studies with the Dengue Virus, another *Flavivirus*, have found the same stop codons in 23% of samples studied. These strains circulated in the population studied for 18 months. These studies suggest that defective genomes can circulate with the assistance of non-defective strains [41,42]. A recent study with Hepatitis C Virus demonstrated that as the virus circulates in the blood, defective genomes can be encapsidated as infectious particles by *trans *complementation (acting on a viral RNA other than the one from which it has been translated) of the structural proteins. NS5A protein was also shown to be the only non-structural protein that acts in *trans *in HCV as well as other *Flavivirus*, so it could be acting as a helper to defective genomes [43-45].

Of the two analytical approaches used in this study, within-sample (*ws*) analysis is relevant when the focus of the study is the patient profile, to study a population, in this case a response group; the statistical power is very low and no or few assumptions can be made. However, between-sample (*bs*) data represent the response group value better, since it is obtained using all 60 sequences from each group. This approach is more accurate when the aim is to identify a pattern from the response group. The *bs *analyses normalize characteristics that are exclusive to individual patients, by using a pool of strains from the same response group. In most cases they yielded statistical significance. Thus, even though the population size of our study is relatively low (12 patients), statistical significance was obtained, supporting the data on the response group patterns discussed.

## Conclusion

This is the first study on the quasispecies composition of the complete NS5A region of HCV genotype 3a. Although we found differences among the response groups, other studies are necessary for a better understanding of the relationship between the variability of this region and the response to treatment with interferon and ribavirin, since most studies are performed with genotype 1, and the genomic differences among these genotypes are significant.

## Methods

### Population and samples

Plasma samples were collected from 12 HCV genotype 3-positive patients, seen at the Hemocenter of São José do Rio Preto, State of São Paulo, Brazil. All samples were collected before treatment, and a 6-months follow-up provided treatment response data. Four patients were sustained virological responders (SVR), i.e the virus was not detected after treatment or during the 6-months follow-up; four were non-responders (NR), since they showed no virological response; and four were end-of-treatment responders (ETR), i.e. a virological response was detected, but at the 6-months follow-up there was a rebound. All patients were infected with HCV genotype 3a. Treatment consisted of INF-α and ribavirin administration for 24 weeks. Patients with a history of alcoholism or infection with another agent that could cause liver damage were excluded. This study was approved by the ethics committee of the Base Hospital of São José do Rio Preto, and all participants signed an informed consent.

### Viral load

The viral load was quantified by Cobas TaqMan HCV Test.

### Extraction of HCV RNA and amplification of the NS5A region

Viral RNA was extracted from blood serum samples using a QIAamp Viral RNA Mini Kit (QIAgen), and cDNA was synthesized using a High-Capacity cDNA Archive Kit (Applied Biosystems) according to the manufacturer's instructions. The NS5A region was amplified using a set of primers specific for genotype 3, designed for this study. For the PCR reaction, two sets of forward and reverse primers were designed. The forward primers were *H.NS5AP-F *(5' GAGCGGTACAGTGGATGAAC 3' - nucleotide [nt] 6089 to 6108 in genotype 3 reference sequence NZL1 - GenBank D17763G) and *H.NS5AP-F2 *(5' GGTACAGTGGATGAACAGG 3' - nt 6093 to 6111 in NZL1). The reverse primers were *H.NS5AP-R *(5' CCTCCTTTAATGCAGTCTTG 3' - nt 7821 to 7840 in NZL1) and *H.NS5AP-R2 *(5' ACGACGTTGAATAGACTAGG 3' nt 7734 to 7753 in NZL1). Two sets of forward and reverse primers were also designed for the nested-PCR reaction. The forward primers were *H.NS5AN-F *(5' CGCATTGCTGAGTTCTCTAAC 3' from nt 6192 to 6212 in NZL1) and *H.NS5AN-F2 *(5' CTCTAACTGTCACAAGTCTGC 3' nt 6206 to 6226 in NZL1). The reverse primers were *H.NS5AN-R *(5' CAACAAGGAGTTGCTGAGTG 3' nt 7703 to 7722 in NZL1) and *H.NS5AN-R2 *(5' CAGCACTACATGGTGTTATC 3' nt 7659 to 7678 in NZL1). For the amplification reaction, 1 U of a proofreading polymerase was used (Elongase^® ^Enzyme Mix; Invitrogen) along with 10 μl of buffer B [300 mM Tris-SO_4_, (pH 9.1 at 25°C), 90 mM (NH_4_)_2_SO_4 _and 10 mM MgSO_4_], 10 μl of dNTP, 30 pmol of sense and anti-sense primers, 10 μl of cDNA for PCR reaction, and 5 μl of PCR product for the nested-PCR reaction, plus Milli-Q autoclaved water to a final volume of 50 μl. The amplified products were analyzed on a 1% agarose gel.

### Cloning and sequencing

Cloning was performed using a TOPO XL Cloning TM Kit (Invitrogen). Fragments from 15 clones from each patient were purified using a PureLink Mini-prep Plasmid Purification Kit (Invitrogen). The entire NS5A region was sequenced using eight primers: the vector primers *M13F *and *M13R*, and six inner primers, three sense and three anti-sense, designed for this study. The forward primers used in the sequencing reaction were: *H.NS5AI-F1 *(5' TGGCTGCGTATCATCTGGGA 3' - nt 6283 to 6302 in NZL1), *H.NS5AI-F2 *(5' ACCTCGATGTTGAGAGACCC 3' - nt 6871 to 6890 in NZL1) and *H.NS5AI-F3 *(5' TATCCTCCAGCCCTTCCTAT 3' - nt 7198 to 7217 in NZL1). The reverse primers used in the sequencing reaction were: *H.NS5AI-R1 *(5' CACGGACACTTGAGCTCATC 3' - nt 6679 to 6698 in NZL1), *H.NS5AI-R2 *(5' TTCTTGAAACACTCTGCAGC 3' - nt 7168 to 7187 in NZL1) and *H.NS5AI-R3 *(5' GTGGACCAAGAGTCGCAACT 3' - nt 7573 to 7592 in NZL1). The sequencing reaction was performed with Dyenamic ET Terminator (GE) and the products were sequenced in an ABI Prism 377 sequencer (Applied Biosystems). The reaction mixture consisted of 1 μl of Milli-Q autoclaved water, 1 μl of primer (5 pmol/μl), 2 μl of sequencing reagent mix, plus 2 μl of sample. Occasionally, when a good quality sequence could not be obtained, the sequencing reaction had to be doubled and a "hot start" had to be performed for 10 min at 95°C for better results. Cycling was carried out according to the manufacturer's instructions.

### Sequence and phylogenetic analysis

The sequences obtained were subjected to BioMol - Electropherogram quality analysis http://adenina.biomol.unb.br/phph/[46], a phred phrap [47,48] analysis site, for quality check and contig construction. The contigs obtained for each clone were aligned, along with the reference sequence NZL1 for genotype 3 (GenBank accession number D17763), using *Clustal X 1.81 *software [49]. All sequences were edited on *Bio Edit 7.0.5.3 *[50] to remove the vector fragments, leaving only the complete sequence of the NS5A region.

Quasispecies analysis was carried out using *LOCSPEQ 1.0 *software [51], specially designed for our group for this kind of analysis.

The number of mutations and the genetic distances were calculated using MEGA 4.0 software [52]. The rates of synonymous substitution per synonymous site (dS) and non-synonymous substitution per non-synonymous site (dN), as well as the dN/dS ratio, were obtained at *SNAP - Synonymous Non-synonymous Analysis Program *-http://www.hiv.lanl.gov[53,54].

Phylogenetic analysis was performed using PAUP* *version 4 *software [55]. A neighbor-joining phylogenetic tree was constructed with Tamura-Nei's substitution model including invariant sites (I) and Gamma distribution shape (G) parameter (TRN+I+G), determined by hierarchical likelihood ratio test score criteria using *Modeltest 3.06 *[56]. One thousand replicates were used to test the reliability of the tree topology, and bootstrap values >70 were considered significant [57].

### Statistical analysis

Statistical analysis was performed by ANOVA. Comparisons between groups were made using Tukey's method for multiple comparisons. Values of P < 0.05 were considered significant. Standard error of the mean (SEM) values were calculated in Minitab 15.

### Types of analysis

In this work, we chose to analyze the results obtained by two different approaches:

#### Within-sample analysis (ws)

This consisted of calculating the means of genetic distances, dS, dN and dN/dS ratios among the clones of one patient, and then calculating the mean of the four values obtained for each patient in the response group to obtain the group value.

#### Between-sample analysis (bs)

This consisted of calculating the means of genetic distances, dS, dN and dN/dS ratios among all the clones of the response group to obtain the group values.

### Nucleotide sequence accession numbers

The nucleotide sequence data reported here have been submitted to the GenBank nucleotide sequence database with accession numbers from EU826174 to EU826352.

## Competing interests

The authors declare that they have no competing interests.

## Authors' contributions

CB, ACGJ, IMVGCM, PR: contributed to the study design, carried out the molecular biology experiments, sequence alignment, phylogenetic analysis and manuscript planning; LHTY: carried out the molecular biology experiments; ATLQ: performed statistical analysis; CMAC: contributed with significant evolutionary knowledge on data analysis; JRRP: contributed to the study design. All authors read and approved the final manuscript.

## Pre-publication history

The pre-publication history for this paper can be accessed here:

http://www.biomedcentral.com/1471-2334/10/36/prepub
